# Evaluation of a communication skills program for first-year medical students at the University of Toronto

**DOI:** 10.1186/1472-6920-9-11

**Published:** 2009-02-22

**Authors:** Solomon M Shapiro, William J Lancee, Christopher M Richards-Bentley

**Affiliations:** 1 Department of Psychiatry, University of Toronto, Centre for Addiction and Mental Health, 250 College Street, Toronto M5T1R8, Canada; 2 Department of Psychiatry, University of Toronto, Mt. Sinai Hospital, 600 University Avenue, 9th floor, Toronto M5G1X5, Canada; 3 Michael G. DeGroote School of Medicine, McMaster University, 1280 Main Street West, Hamilton L8S4L8, Canada

## Abstract

**Background:**

Effective doctor-patient communication has been linked to numerous benefits for both patient and physician. The purpose of this study was to evaluate the effectiveness of the University of Toronto's Therapeutic Communication Program (TCom) at improving first-year medical students' communication skills.

**Methods:**

Data were collected during the 1996/97, 1997/98, 1998/99 and 1999/00 academic years. The study used a repeated measures design with a waiting list control group: students were randomly assigned to groups starting the educational intervention in either September (N = 38) or February (N = 41), with the latter being used as a control for the former. Communication skills were assessed at the pre- and post-intervention times and at the end of the academic year from the perspectives of student, standardized patient and external rater.

**Results:**

Only the external rater, using an instrument designed to assess the students' empathy based on their written responses, showed a time × group interaction effect (p = 0.039), thereby partially supporting the hypothesis that TCom improved the students' communication skills. Students rated themselves less positively after participation in the program (p = 0.038), suggesting that self-evaluation was an ineffective measure of actual performance or that the program helped them learn to more accurately assess their abilities.

**Conclusion:**

The lack of strong findings may be partly due to the study's small sample sizes. Further research at other medical or professional schools could assess the effectiveness of similar courses on students' communication skills and on other capacities that were not measured in this study, such as their understanding of and comfort with patients, their management of the doctor-patient relationship, and their ability to give and receive feedback.

## Background

Effective doctor-patient communication has been linked to numerous benefits, including patient recall and understanding, adherence, symptom resolution, reduction in psychological distress, perception of physician competence, and patient and physician satisfaction. [1-4] Furthermore, several studies as well as licensing bodies have found that poor communication is the most frequent underlying cause of complaints against physicians and malpractice allegations. [5,6]

The Therapeutic Communication Program (TCom), launched by the Department of Psychiatry at the University of Toronto in 1995, was loosely modelled after the Student Psychotherapy Scheme, an elective program offered at the University College Hospital in London since 1958. [7] TCom offers first-year medical students the opportunity to meet weekly with patients on a one-to-one basis for four months while receiving group supervision from a faculty psychiatrist.

The program seeks patients who are relatively healthy psychologically, have one or two psychosocial issues they would like to address, have a good network of support, and are sufficiently motivated and committed to attend three months of weekly sessions. Common presenting problems are losses (e.g., deaths, loss of health, loss of jobs), relationship difficulties, problems at work and impending life decisions. The screening process has three levels: First, an attempt at self-selection is made by underscoring the importance of focusing on a key issue and willingness to commit to regular attendance in promotional materials. Second, the program's intake coordinator conducts a telephone interview involving key questions designed to assess the nature and scope of the patient's issue as well as his or her motivation, support structure and risk of self-harm. Callers for which TCom is deemed unsuitable are encouraged to seek help elsewhere and are provided with alternate resources when appropriate. Third, the supervising psychiatrist and the other 3–4 students in his or her TCom group observe and evaluate the student's intake assessment with the patient, allowing difficulties to be identified and addressed if they arise. While it sometimes emerges over the course of the sessions that a patient has problems and interpersonal patterns that were not evident during the phone interview or intake assessment, to date there have not been difficulties or crises that could not be handled by the student.

The supervisor conducts weekly meetings with his or her TCom students. He or she aims to promote a sense of safety in the group supervision so that students feel free to share and explore their personal reactions to patient sessions; at the same time, supervisors ensure that, in the process, the line is not crossed that would turn supervision into psychotherapy. With the goal of teaching students to seek and provide meaningful collegial support, supervisors encourage students to offer each other constructive feedback and suggestions.

Schonfield and Donner [8] question the value of exposing all medical students to the psychotherapist's role, finding that "technique-oriented" medical students develop more negative views of their patients and of their own efficacy than "person-oriented" medical students. TCom aims to minimize student disappointment and risk of patient harm through a focus on the student-patient relationship during the program and informed student self-selection beforehand: the program is offered to students on a voluntary basis and they are provided with a careful description of the program's expectations and challenges. Potential student participants are told that the program strives to accomplish three goals: first, to increase the ability of the students to interact effectively with medical patients; second, to strengthen their skills in eliciting, understanding and utilizing the various types of psychological information available in a doctor-patient relationship; and, third, to enhance their curiosity, tolerance and comfort in dealing with a variety of patients and with different kinds of symptoms, emotions, attitudes and behaviours.

Aspegren [9] suggests that instructional methods of communication skills training (i.e., those involving the teacher demonstrating or lecturing on how to conduct an interview and the student repeating the skill with or without feedback) are ineffective when compared with experiential methods, where the student does the interview him- or herself and then receives feedback from the teacher. However, the literature is not clear regarding how best to teach communication skills, such as for how long or whether different students require different durations or types of training. [10-13] Furthermore, many studies are flawed by a lack of control group. [14,11] Finally, the literature is problematic in that evaluation of these programs and their influence on one's own communication skills learning is frequently based on students' satisfaction ratings; [11] students' self-perception of ability does not necessarily correlate highly with other measures, such as standardized examinations and evaluations of the students given by the faculty, and this commonly results in lower-performing students overrating themselves. [15,16]

The purpose of this study was to assess the effectiveness of TCom in improving first-year medical students' communication skills, which, for the purpose of this study, were defined as the ability to: (a) engage someone in a conversation; (b) maintain a conversation; (c) understand another person's perspective; (d) accurately track the emotional state of the other; (e) express care and concern without intrusiveness or use of platitudes; (f) do all of the above without negating, belittling, or being controlling; (g) elicit relevant information in an efficient manner (e.g., stay on topic); and (h) explain and describe clearly and succinctly. It was hypothesized that, compared to waiting list control participants, group supervision participants would have a greater improvement in their therapeutic communication skills over the time of the intervention.

## Methods

Over four consecutive academic years, beginning in the 1996/97 academic year, funding from the Medical Research Council of Canada (now the Canadian Institutes of Health Research) and the Association of Canadian Medical Colleges was granted to test TCom. Ethics approval was granted by the University of Toronto Department of Psychiatry.

### Experimental Design

**Figure 1:**
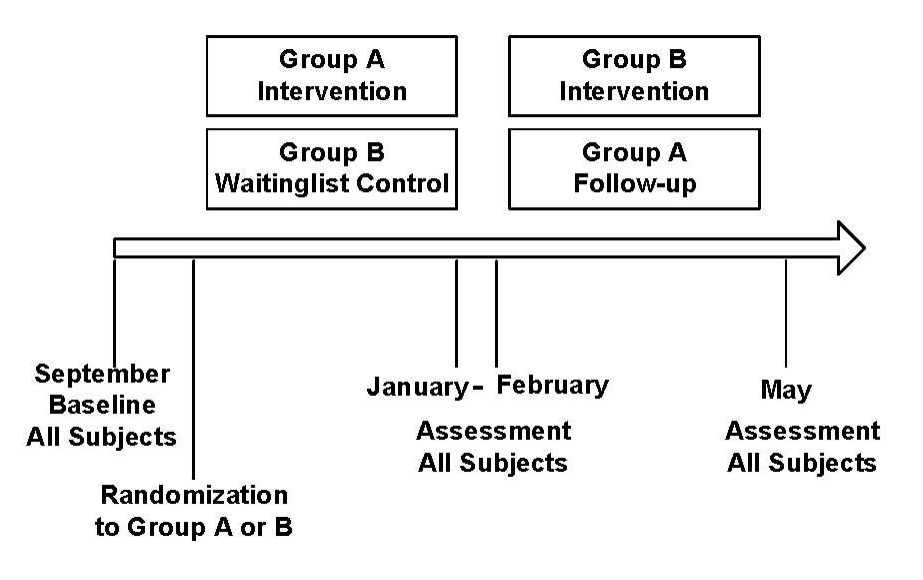
Experimental Design (Randomized Controlled)

**Figure 2:**
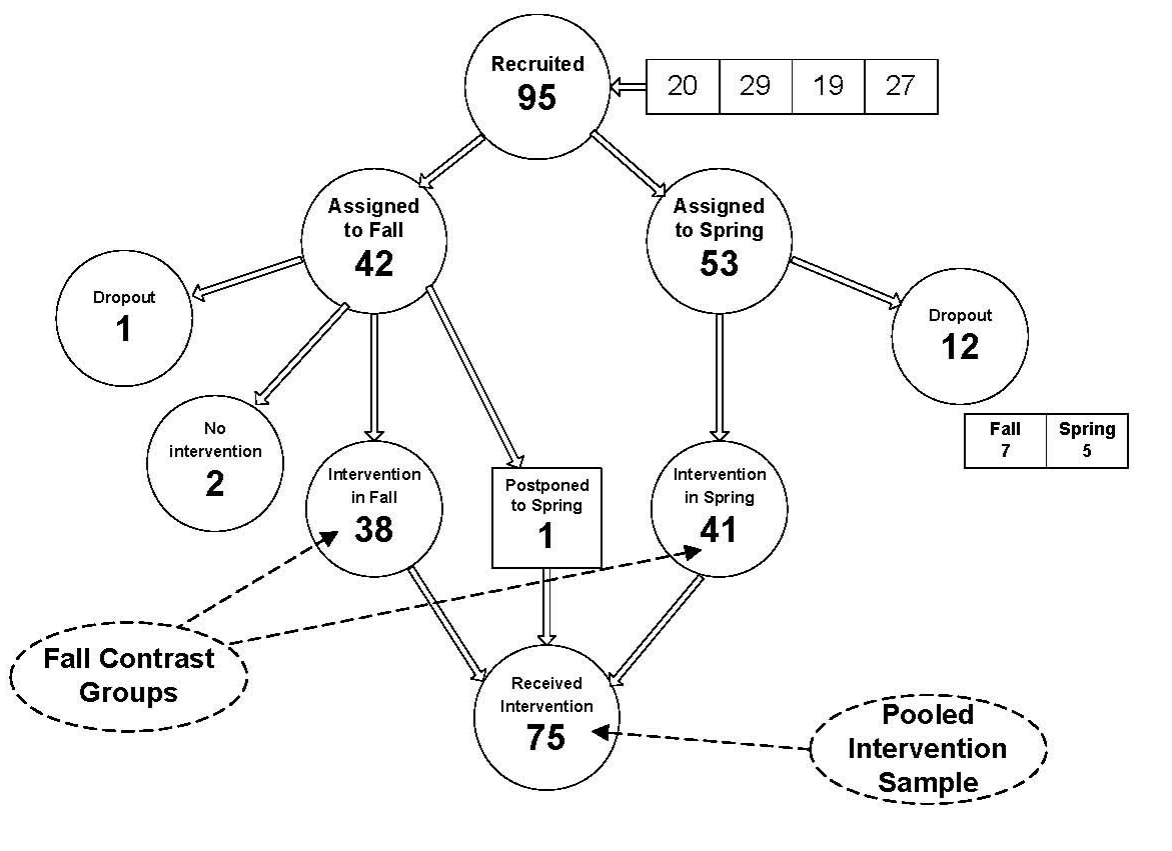
Participant Flow

The design was a repeated measures with a waiting list control group. After providing informed written consent and completing the screening procedure, students were randomly assigned to groups starting the educational intervention in either September or February. The students starting in February were used as the waiting list control group for the students starting in September (the intervention participants). The original target samples were 40 intervention participants and 40 control participants. Data were collected over four academic years, yielding 38 intervention participants and 41 control participants. The assessment protocol was administered at baseline, at the end of the intervention (four months later), and at the end of the academic year (approximately eight months after baseline). Figure 1 shows the design of the study. Figure 2 shows the total number of participants (students participating in TCom over the period of the study).

### Instruments

Communication skills were assessed at the pre- and post-intervention times and at the end of the academic year from three perspectives: (a) student; (b) standardized patient (actor); and (c) external rater:

(a) The participants completed the Self Assessment of Interpersonal Competence Questionnaire (SAICQ), [17] a 40-item scale, with a Likert scale of 1 (I'm poor at this) to 5 (I'm extremely good at this). Examples of items are: "Carrying on conversations with someone new whom you think you might like to get to know" and "Turning down a request by a companion that is unreasonable".

(b) Participants interviewed two standardized patients (SPs), [18] actors portraying patients with psychosocial problems, trained and tested for reliability by the Department of Family and Community Medicine Standardized Patient Program of the University of Toronto. The interviews were rated by the SPs using the Interpersonal Skills Rating Scale (ISRS). [19] There are 7 items, with a Likert scale of 1 (strongly disagree) to 7 (strongly agree). Examples are: "The doctor wanted to understand how I saw things" and "The doctor just took no notice of some things that I thought or felt".

(c) The Staff-Patient Interaction Rating Scale (SPIR), [20,21] a reliable (test-retest r = .79) and valid (φ = .67–.78) instrument designed to assess participants' expressed empathy based on their written open-ended responses to a series of 24 statements made by hypothetical patients, was administered. Examples of statements are: "Why do I have to keep on seeing you?" and "I just want to do nothing and stay in bed". The responses to these items were rated by trained external raters according to a manual that describes in detail how to classify responses into disengaging and engaging sets. The score for each of the 24 items on the scale ranges from -1 for a disengaging response, 0 for a neutral response, to +1 for an engaging response. Responses were scored by raters who were blind to the randomization, the identity of the participant and concurrent other responses by a participant. On average, 10 out of the 24 responses can be expected to be neutral. Improvements are demonstrated by an increase in engaging responses and a decrease in disengaging responses.

The conclusions drawn from the three measures were assessed alongside the results from satisfaction questionnaires that were completed anonymously by TCom students during the 2000/01, 2001/02 and 2002/03 academic years.

## Results

Table 1 shows the basic characteristics of the sample and demonstrates that the two contrast groups were comparable. The mean age and gender distributions were as expected, being comparable to those for the first-year medical student class at the University of Toronto. One-third of the sample was not born in Canada and only one-tenth of participants had a formal background in psychology. At baseline, the intervention and control groups were equivalent with respect to therapeutic skills competency levels from all three perspectives. It should be noted that, at baseline, the SPIR scores correlated significantly (at the p < .01 level) with both self and actor ratings (r = .31 and r = .29, respectively). However, the actor and self perspectives did not correlate significantly (r = .15).

**Table 1:** Characteristics of Participants at Baseline by Randomization Group.

	All Subjects	Intervention	Control Subjects	Significance
	n = 79	n = 38	n = 41	(N.S. = not significant)
Gender				
Female	61%	63%	59%	Π2 (1) = 0.18
Male	39%	37%	41%	N.S.

Age	23.1	23.1	23.0	F(1,77) = 0.03
(S.D.)	(2.59)	(2.61)	(2.61)	N.S.

Education				
B.Sc.	72%	66%	78%	Π2 (1) = 2.10
Grad Biology	19%	21%	17%	N.S.
Psychology	9%	13%	5%	Π2 (1) = 1.43

Canadian born	67%	61%	73%	N.S.

Table 2 presents the results of the test of the hypothesis. Using a multivariate (incorporating all three perspectives), there was no significant overall time × group interaction effect, where "time" denotes the duration between testing at baseline and testing four months later. In other words, the changes over time in the intervention were not distinct from the changes in the control group. However, there was a significant measure × time × group interaction effect, indicating the possibility of one or more measures showing the hypothesized time × group interaction effect. As can be seen from the rows in Table 2, only the SPIR showed a significant time × group interaction effect, thereby supporting the hypothesized intervention effect.

**Table 2:** Mean Pre-Post Values and Changes in Outcome Variables in Experimental Phase.

	Intervention			Control			Significance (N.S. = not significant)
	(A)	(B)	(B) - (A)	(A)	(B)	(B) - (A)	Repeated
	pre	post	change	pre	post	change	Measures
							Time × Group

Self Rating, SAICQ	3.39	3.31	-0.09	3.54	3.57	+0.02	F(1,77) = 2.82
(S.D.)	(0.61)	(0.56)	(0.29)	(0.46)	(0.45)	(0.28)	N.S.

Actor Rating, ISRS	59.7	65.3	+5.7	58.2	61.1	+2.9	F(1,77) = 0.54
(S.D.)	(13.4)	(14.2)	(16.8)	(13.0)	(14.0)	(16.8)	N.S.

External Rating, SPIR	6.00	8.29	+2.29	7.32	6.63	-0.68	F(1,77) = 4.46
(S.D.)	(5.72)	(4.98)	(6.30)	(6.68)	(5.13)	(6.20)	p = 0.038

Three-Measure Multivariate Time × Group F(1,77) = 1.90, not significant. Measure × Time × Group F(1,77) = 4.85, p = 0.03.

All participants were pooled for the evaluation of change over the academic year, after the control participants also received the intervention. The results are shown in Table 3. While the SAICQ measure shows a significant decrease over time, the ISRS and SPIR measures show a significant improvement over time. There was no time × cohort interaction effect, demonstrating that both cohorts of participants (Fall intervention and Spring intervention students) show effect. Finally, compared to baseline, 50% of participants improved by decreasing their disengaging responses (by at least two out of 24 SPIR items) or by increasing their engaging responses (by at least two out of 24 SPIR items).

**Table 3:** Mean Pre-Post Values and Changes in Outcome Variables in Open Phase (Assessment Data Available Varies from n = 71 to n = 73).

	Intervention over Academic Year			Significance
	(A)	(B)	(B) - (A)	Repeated Measures
				Time Effect

Self Rating, SAICQ	3.47	3.39	-0.08	F(1,70) = 4.45
(S.D.)	(0.55)	(0.55)	(0.32)	p = 0.038

Actor Rating, ISRS	59.1	65.5	+6.4	F(1,69) = 11.38
(S.D.)	(13.1)	(12.7)	(16.0)	p < 0.001

External Rating, SPIR	6.70	8.33	+1.63	F(1,71) = 4.43
(S.D.)	(6.15)	(5.51)	(6.58)	p = 0.039

Three Measure Multivariate Time F(1,66) = 17.28, p < 0.001. Time × Group F(1,66) = 0.18, not significant.

## Discussion

While the hypothesis was supported by only the SPIR ratings, the results from Table 3 suggested that this was partly due to the low power of small sample sizes to test the contrast between intervention participants and controls. It should be noted that the 12 participants who lost interest while on the waiting list were not included in the above analysis as intend-to-treat participants because their inclusion as no-change controls would inflate the contrast between the two groups.

Because this study employed three methods of evaluating medical students' communication skills, the results may contribute to the discussion regarding which evaluation methods are most productive. The fact that students rated themselves less positively after the experimental manipulation suggests that either TCom was detrimental to students' ability to therapeutically communicate or that students' self-evaluation of communication skills is an ineffective measure of actual performance. The second of these conclusions is supported by the fact that the actor and self perspectives did not correlate significantly (r = .15). However, Boud and Lublin [22] state that becoming an accurate self-evaluator and developing the ability to monitor one's own learning process represents "one of the most important processes that can occur in undergraduate education", and this may be especially true for medical students who, once working as practicing physicians, will need to rely primarily on self-evaluation to monitor their performance. Therefore, training students to be accurate self-evaluators should be viewed as an important goal of medical school education, one that may be achieved partly through programs such as TCom. Given that Boud and Falchikov [23] identify the general trend for high-achieving students to underestimate their abilities while low-achieving students overestimate theirs, the finding that students decreased their self-evaluations as a result of TCom could itself be indicative of meaningful learning in that the students' self-evaluations became more accurate. While students were not identified as either high-achieving or low-achieving upon entering the study, making it impossible to confirm whether the experimental manipulation caused low-achieving students to lower their initially-high ratings and high-achieving students to raise their initially low ratings, the fact that all participants were first-year medical students with the majority coming from a science background suggests that most would initially be classified as low-achieving in the domain of communication, and that an overall decline in the SAICQ occurs as their self-evaluations become more accurate.

One may ask, however, whether the decrease in students' self-evaluations could correspond to feelings of discouragement, as this could decrease the likelihood that they would pursue such learning opportunities in the future. An answer to this question may be inferred from the data provided by anonymous program evaluation forms completed by TCom students during the 2000/01, 2001/02 and 2002/03 academic years. Sixty of the 81 TCom students who participated in TCom (74%) completed and returned the questionnaires. While 21 of the 254 (8%) comments made by students involved their difficulty with managing patient encounters, all except one student (98%) felt that they benefited from their experience in TCom, 78% of them substantially. Fifty-five students (92%) indicated that the time expenditure was worthwhile and fifty (83%) said that they would recommend the program to other students. Specifically, fifty students (83%) reported improvement in the way they listen and talk to patients. There were no significant differences in student responses across the three years. Because students generally rated their experiences in TCom very favourably, it is unlikely that the decrease in their self-evaluations was based on feelings of discouragement and more likely that this indicates an improvement in their accuracy as self-evaluators.

The finding that self-perceptions did not correlate significantly with actor perceptions (r = .15) also indicates the need for medical students to get intensive supervision of their clinical work early in their medical training. TCom students generally rated the supervised group format very positively. Fifty-four (92%) of the 59 students who evaluated their TCom supervisors rated them as, overall, very good to excellent. Students rated their supervisors as excellent in the areas of organization, providing a positive learning environment, and providing helpful direction and feedback.

While the students generally rated their supervisors very positively, fifty-four respondents (92%) expressed the wish for more guidance from them. However, despite this feedback, the program coordinator and supervisors elected to continue offering minimal advice, considering it essential that students discover their own interactional style and learn to give and receive collegial support and feedback. Indeed, students often reported that they became more self-aware and open with fellow students, and described the supervision group as a safe venue for discussing challenging situations and for learning from each other.

While the study's final assessment protocol was administered approximately eight months after baseline, one might still question whether the medical students' communication skills learning was maintained over a greater length of time. While the assessment of longer-term reinforcement was beyond the study's scope, other research regarding the long-term reinforcement of communication skills has suggested that communication skills may be retained over longer periods of time. Bowman et al. [24] assessed the psychiatric interview skills of physicians 18 months after they attended a problem-based interviewing course and reported that "not only were acquired skills maintained but further changes took place during the follow-up period, change that can be seen as improvement in terms of the course model." Furthermore, a study by Maguire, Fairbairn and Fletcher [25] found lasting improvement in communication skills into residency. Therefore, while our study does not assess the retention of communication skills over a period longer than four months, it is hoped that any learning afforded by TCom will benefit the program's medical students throughout the rest of their education and as practicing physicians.

It is also possible that, while the SPIR ratings confirmed the hypothesis, the SAICQ and ISRS failed to confirm the hypothesis specifically because they were unable to identify the factors in which students may have actually improved, such as their ability to form, develop and maintain therapeutic relationships over time. It is possible that the structure of TCom, where training occurs over several months, is ideal for the development of such skills, and that a different measure would have to be used in order to determine whether TCom is successful in attaining its goals. Another possible interfering factor might be an overestimation of student skills by the standardized patients, reflecting a wish to see the students in a positive light (i.e., to give them "the benefit of the doubt").

## Conclusion

The hypothesis that the training provided by the Therapeutic Communication Program (TCom) improved the students' communication skills was supported by the externally rated SPIR instrument but not by the other two measurement perspectives. Based on the SPIR, participants in TCom, even those without a psychology background, increase their engaging responses and decrease their disengaging responses in hypothetical challenging clinical encounters. The continuous operation of TCom at the University of Toronto, despite a lack of research to date clearly demonstrating its educational effectiveness, is a result of very positive ratings of the program by students and by their teachers and patients. Further research at different medical schools and other professional schools could be used to assess the effectiveness of similar programs on communication and these other capacities. Longitudinal studies could assess the maintenance of enhanced skills over longer periods of time.

## Competing interests

The authors declare that they have no competing interests.

## Authors' contributions

SS developed the Therapeutic Communication Program and has coordinated it since its inception. WL conceived of and designed the study. SS participated in the design of the study. WL and SS participated in the coordination of the study. WL performed the statistical analysis. All authors participated in the evaluation and interpretation of the study results. All authors drafted the manuscript. All authors read and approved the final manuscript.

## Authors' Information

**Dr. Solomon M. Shapiro **is an Assistant Professor of Psychiatry at the University of Toronto and a staff psychiatrist at the Centre for Addiction and Mental Health. His areas of expertise include child and adolescent psychiatry; the teaching of communication skills to medical students; individual, family and couple therapy; and the teaching of psychotherapy. Dr. Shapiro developed in 1995, and continues to head, the Therapeutic Communication Program, which offers first-year medical students the opportunity to meet weekly with a patient on a one-to-one basis while receiving group supervision from a faculty psychiatrist. The program strives to increase the students' ability to interact effectively with patients.

**Dr. William J. Lancee **is an Associate Professor of Psychiatry at the University of Toronto whose research carries a special emphasis on the quality of communication between patient and care-provider. He is the creator and developer of the Wellness Interview, a web-based computerized interview that maps client answers to a report that is meaningful to health care providers. He is also an investigator in a funded project to improve doctor-patient communication in difficult patient situations. Dr. Lancee has degrees in health research as well as in mathematics and computer science. He has expertise in statistics, instrument development, epidemiology, program evaluation, and research methodology.

**Christopher M. Richards-Bentley **is a medical student at the Michael G. DeGroote School of Medicine at McMaster University, Hamilton. He volunteered as the Therapeutic Communication Program's Intake Coordinator from 2006–2008.

## Pre-publication history

The pre-publication history for this paper can be accessed here:


